# Precise prediction of hotspot residues in protein–RNA complexes using graph attention networks and pretrained protein language models

**DOI:** 10.1093/bioinformatics/btaf197

**Published:** 2025-07-15

**Authors:** Siyuan Shen, Jie Chen, Zhijian Huang, Yuanpeng Zhang, Ziyu Fan, Yuting Kong, Lei Deng

**Affiliations:** School of Computer Science and Engineering, Central South University, Changsha, 410083, China; School of Software, Xinjiang University, Urumqi, 830046, China; School of Computer Science and Engineering, Central South University, Changsha, 410083, China; School of Software, Xinjiang University, Urumqi, 830046, China; School of Computer Science and Engineering, Central South University, Changsha, 410083, China; School of Information Engineering, Xinjiang Institute of Engineering, Urumqi, 830023, China; School of Computer Science and Engineering, Central South University, Changsha, 410083, China

## Abstract

**Motivation:**

Protein–RNA interactions play a pivotal role in biological processes and disease mechanisms, with hotspot residues being critical for targeted drug design. Traditional experimental methods for identifying hotspot residues are often inefficient and expensive. Moreover, many existing prediction methods rely heavily on high-resolution structural data, which may not always be available. Consequently, there is an urgent need for an accurate and efficient sequence-based computational approach for predicting hotspot residues in protein–RNA complexes.

**Results:**

In this study, we introduce DeepHotResi, a sequence-based computational method designed to predict hotspot residues in protein–RNA complexes. DeepHotResi leverages a pretrained protein language model to predict protein structure and generate an amino acid contact map. To enhance feature representation, DeepHotResi integrates the Squeeze-and-Excitation (SE) module, which processes diverse amino acid-level features. Next, it constructs an amino acid feature network from the contact map and SE-module-derived features. Finally, DeepHotResi employs a graph attention network to model hotspot residue prediction as a graph node classification task. Experimental results demonstrate that DeepHotResi outperforms state-of-the-art methods, effectively identifying hotspot residues in protein–RNA complexes with superior accuracy on the test set.

**Availability and implementation:**

The source code and dataset are available at https://github.com/Q1DT/DeepHotResi.

## 1 Introduction

Protein–RNA interactions play pivotal roles in various biological processes, such as DNA repair, RNA splicing, protein synthesis, and gene regulation. Recent studies have emphasized the critical involvement of specific protein–RNA complexes in diseases, including cancer (Gebauer *et al.* 2021) and neurological disorders (Huarte *et al.* 2010). Aberrant expression or functional alterations in these complexes are closely associated with disease onset. Experimental evidence (Meenan *et al.* 2010) indicates that modifications to just a few critical residues (e.g. 3–4 residues at the E9 DNase-Im9 immunity protein interface) can substantially alter binding specificity. These findings suggest the existence of synergistically interacting residues, termed *energy hotspot clusters*. In protein–RNA interfaces, although binding sites typically span extensive regions, certain individual residues—known as hotspot residues—significantly influence the overall binding free energy (ΔΔG). These residues are essential targets in drug design (Clackson and Wells 1995), as precisely targeting hotspot residues can facilitate the development of more selective small-molecule drugs, potentially reducing undesirable side effects (Cukuroglu *et al.* 2014). Currently, hotspot identification primarily depends on alanine scanning mutagenesis and thermodynamic measurements of binding energy changes. Nevertheless, these experimental methods are labor-intensive, expensive, and prone to inconsistent results due to inherent experimental variability (Wells 1991). Consequently, developing rapid, accurate computational approaches for predicting hotspot residues in protein–RNA complexes is imperative for advancing drug discovery and therapeutic strategies.

Advancements in bioinformatics have led to continuous progress in computational methods for hotspot residue prediction, which generally fall into three categories:

- *Molecular simulation-based methods*: Molecular dynamics simulations were initially employed to mimic alanine substitution, estimating corresponding changes in binding free energy (Massova and Kollman 1999, Brenke *et al.* 2009). Although numerous simulation-based techniques have successfully identified hotspot residues (Huo *et al.* 2002), they incur high computational costs, limiting their scalability for large-scale predictions.
- *Knowledge-based methods*: These methods leverage established principles from physics and chemistry, enhancing interpretability. For instance, Guerois *et al.* (2002) introduced FOLDEF, leveraging the EEEF energy function to predict hotspot residues. Kortemme and Baker (2002) proposed Robetta, an empirical method utilizing simplified physical models to estimate binding energies. Although knowledge-based methods significantly improve efficiency, they often exhibit limitations regarding adaptability to complex systems and prediction accuracy.
- *Machine learning-based methods*: Recently, machine learning techniques have increasingly been applied to hotspot prediction. Barik *et al.* (2016) developed HotSPRing, focusing on evolutionary conservation, structural, and physicochemical properties of protein–RNA interfaces. Using a random forest classifier, they accurately predicted approximately 80% of binding free energy variations. Pan *et al.* (2018) introduced PrabHot, an ensemble method utilizing conceptually diverse machine learning classifiers. Employing the ensemble voting classifier alongside the Boruta algorithm, PrabHot identified 35 optimal features derived from sequence, structural, and interaction network-based properties, outperforming earlier predictors. Deng *et al.* (2019) proposed XGBPRH, combining extreme gradient boosting (XGBoost) with a two-step feature selection process to determine six optimal attributes from sequence, structural, solvent exposure, and interaction network properties, achieving remarkable performance in both F1 scores and ROC curves. Recently, Zhou *et al.* (2022) developed SREPRHot, utilizing the SMOTE algorithm and introducing a novel random grouping feature selection technique to extract crucial features from sequence and structural information, enhancing prediction accuracy through stacking ensemble classifiers.

Despite these advances, the limited availability of experimental datasets substantially hampers research into predicting hotspot residues in protein–RNA complexes. Additionally, most existing computational methods heavily depend on structural data, constraining their broader applicability and accuracy. Therefore, novel approaches that can effectively overcome these limitations are urgently required.

In this study, we introduce DeepHotResi, a novel sequence-based method for predicting hotspot residues in protein–RNA complexes. Unlike previous methods, DeepHotResi requires only the protein sequence as input, eliminating the reliance on complex structural data. The method consists of three main stages: (i) predicting the 3D structure of the protein using the pretrained evolutionary sequence model 2 (ESM-2) model to generate contact maps; (ii) integrating protein sequence and structural features, such as hidden Markov model (HMM) features, define secondary structure of proteins (DSSP) features, position-specific scoring matrix (PSSM) features, and embeddings from the pretrained protein language model. These features are then processed using the squeeze-and-excitation (SE) module to recalibrate and enhance the representation of protein residues; (iii) using a graph attention network (GAT) to predict hotspot residues in protein–RNA complexes. To address the challenge of limited hotspot residue data, we collect a new dataset containing 311 hotspot residues from 114 protein–RNA complexes (99 complexes allocated for training and 15 for testing), manually curated from literature and databases. Additionally, the dataset is augmented using a label transfer method. Experimental results show that DeepHotResi achieves high accuracy in hotspot residue prediction and outperforms existing state-of-the-art methods.

## 2 Materials and methods

### 2.1 Datasets

We constructed a training dataset to facilitate the prediction of hotspot residues in protein–RNA complexes. This dataset consists of mutational energy data for 835 residues derived from 147 protein–RNA complexes. Data were collected from multiple sources, including published literature, the Nabe database (Liu *et al.* 2021), prior relevant studies (Barik *et al.* 2016, Pan *et al.* 2018, Deng *et al.* 2019, Zhou *et al.* 2022), and supplementary data generated through a label transfer method (detailed in Section 2.7). A comparison of dataset sizes is provided in Table 1. To minimize redundancy, proteins with sequence similarity greater than 40% were removed using the cluster database at high identity with tolerance. Residues exhibiting a binding free energy change (ΔΔG) of ≥1.0 kcal/mol were classified as hotspots. The independent test dataset, consisting of 15 protein–RNA complexes, was sourced from our previously published work (Pan *et al.* 2018). To eliminate potential data leakage, sequences within the training set were further screened using pairwise alignment to ensure they shared no significant sequence similarity (>40% identity) with sequences in the test dataset. As a result, the final training dataset encompasses 99 protein–RNA complexes, containing 283 hotspot residues and 368 nonhotspot residues.

**Table 1.** Comparison of dataset sizes for hotspot residues prediction of protein–RNA complexes.

			Hotspot residues defining threshold	
Dataset name	No. of proteins	No. of residues	ΔΔG≥1.0 kcal/mol	ΔΔG≥2.0 kcal/mol
Zhou *et al.*’s dataset	58	229	119	45
Pan *et al.*’s dataset	47	209	107	29
Barik *et al.*’s dataset	13	80	26	4
Our dataset	**114**	**709**	**311**	**65**

Note: The bold values indicate the largest number of proteins and hotspot residues among the listed datasets.

### 2.2 DeepHotResi architectures

**Figure 1.**
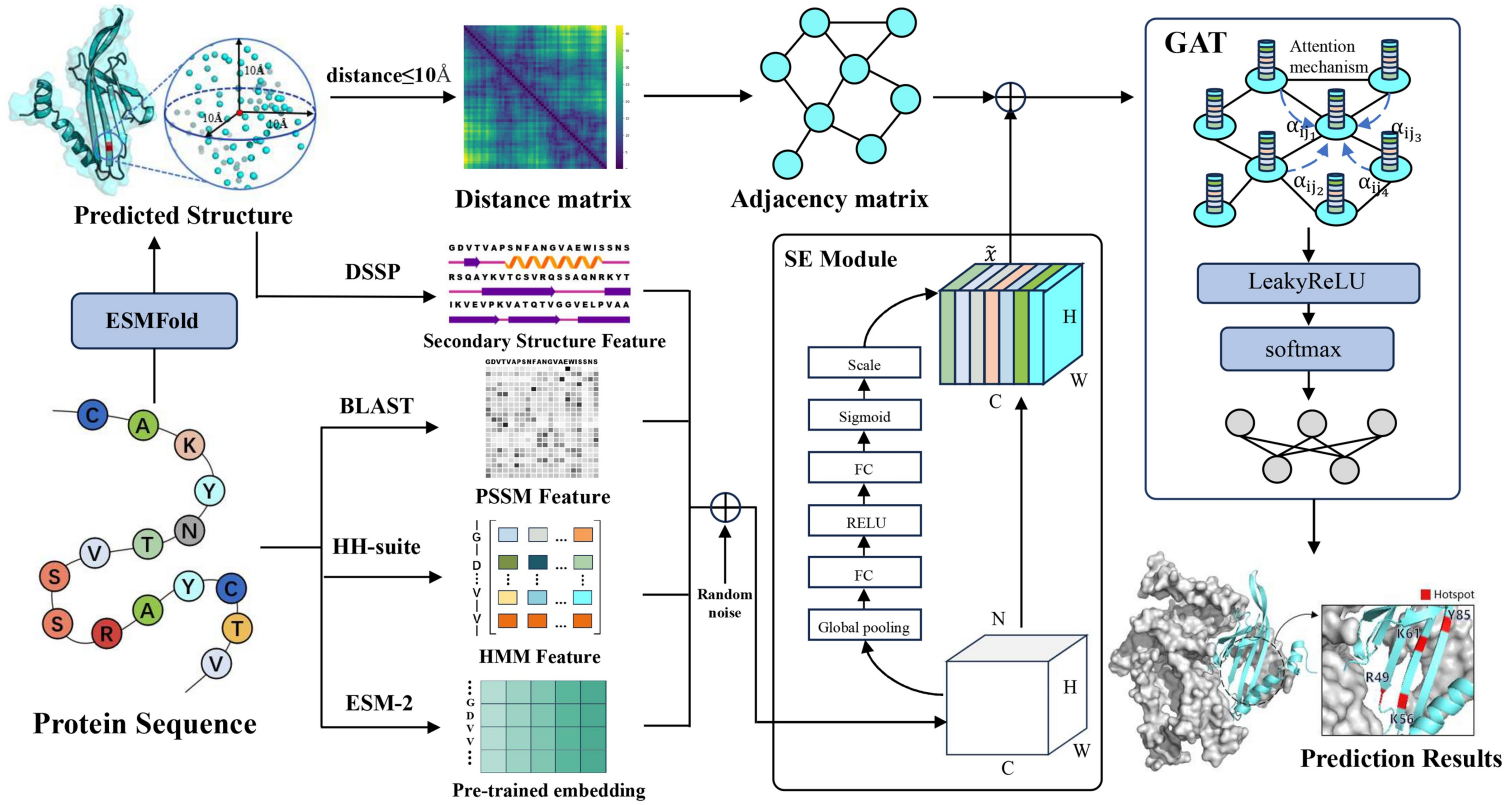
Flowchart of the DeepHotResi model. The model takes the protein sequence as input, predicts its 3D structure using the ESM-2 pretrained model, and constructs a contact graph. It then extracts various features from both the sequence and structure, including HMM, DSSP, and PSSM features, as well as sequence embeddings from pretrained language models. After integrating these features, the model applies SE-NET for feature enhancement and recalibration. Finally, hotspot residues are predicted using the GAT.

In this study, we propose a deep learning framework, DeepHotResi, designed to predict hotspot residues in protein–RNA complexes. Unlike traditional methods that rely heavily on complex structural data, DeepHotResi requires only the protein sequence as input. An overview of the proposed method is illustrated in Fig. 1. Initially, the ESM-2 pretrained model is used to predict the structural information of the protein, from which a contact map is generated. Next, we extract features from both the structural and sequence data: structural features are derived using DSSP, while sequence features and evolutionary information are obtained through HMM and PSSM. Additionally, the ESM-2 model is used to compute embeddings for each residue. To further enhance feature representation, the SE-module is employed for feature recalibration, optimizing the model’s ability to capture key information. Finally, the proposed method applies GAT to aggregate the features of adjacent nodes and edges, allowing DeepHotResi to accurately predict hotspot residues in protein–RNA complexes. The attention mechanism in the GAT highlights residues that have the greatest impact on the prediction, thereby improving the model’s interpretability.

### 2.3 Protein contact map

ESM-2 is used to predict protein structure and construct a protein contact map. In this process, each residue is regarded as a node, and the alpha-carbon atom is chosen to calculate the pseudoposition representation of the residues. By calculating the Euclidean distance between residues, we set a distance threshold of 10 angstroms (Å). If the distance between nodes of two residues was <10 Å, an edge was considered to exist between these two nodes. Regarding the setting of the residue distance threshold, we refer to several recommended values in the literature (Boadu *et al.* 2023), and select 6, 8, 10, and 12 Å as different thresholds for experimentation. The experimental results show that the model performs best when the residue distance threshold is set to 10 Å. The impact of the protein contact map is described in the Supplementary Material.

### 2.4 Protein representations

The proposed method utilizes a combination of features for residue representation, including secondary structures, PSSM, HMM, and pretrained embeddings generated by ESM-2.

#### 2.4.1 Secondary structure feature

DSSP (Kabsch and Sander 1983) is used to classify each amino acid residue in the protein into secondary structure types such as α-helix, β-strand, or irregular coil. In our model, the secondary structure feature dimension is 14, encompassing not only standard secondary structure types but also finer classifications like β-bridge and turn.

#### 2.4.2 PSSM features

PSSM (Jones 1999) features are created by aligning a multitude of homologous sequences to reflect the relative frequency of different amino acids at each position. In our model, the dimensionality of the PSSM feature is 20, meaning that each position in the sequence is represented by a vector of 20 elements, each corresponding to the occurrence probability of the 20 standard amino acids. PSSM not only reveals the frequency of amino acids, but also indicates the evolutionary pressure at specific positions of the protein.

#### 2.4.3 HMM features

HMM (Eddy 1996) is a statistical model for modeling sequence data, which is widely used in the analysis of protein, DNA and RNA sequences. In protein sequence analysis, HMM features can reveal complex patterns and relationships in amino acid sequences. The dimension of the HMM features used for residue representation in our model is 20.

#### 2.4.4 Language model representation

ESM-2 (Lin *et al.* 2023) is a fast and accurate end-to-end protein language model that is good at using evolutionary and sequence information to generate protein features. This representation not only contains protein sequence information but also captures their interaction information in 3D space. This study uses the sequence-based 3B parameter language model in the UniRef50 dataset (Suzek *et al.* 2007) and uses ESM-2 to extract a 2560-dimensional sequence embedding for each residue.

### 2.5 Processing features using SE-module

Squeeze-and-Excitation Networks (SE-NET) (Hu *et al.* 2018) are a core component of our model, employed to enhance the feature representation of residues. SE-NET leverages global information to selectively emphasize features with high informational content while suppressing less useful ones (Niu *et al.* 2021). This is achieved by automatically adjusting the importance of each channel feature through an information exchange process. The operation of SE-NET involves two primary steps: squeeze and excitation. The specific formulas for these steps are as follows:


(1) $$Z=F_{\text{sq}}(u_{c})=\frac{1}{H\times W}\sum_{i=1}^{H}\sum_{j=1}^{W}u_{c}(i,j)$$

(2) $$S=F_{\text{ex}}(Z,W)=\sigma (W_{2}\delta (W_{1}Z))$$

Here, $u_{c}$ represents the feature map of a specific channel *c*, and *H* and *W*, respectively, denote the height and width of the input feature map. δ is the LeakyReLU function; σ is the sigmoid function, with weights between 0 and 1. The reduction ratio *r* in our model is set to 16, which helps to balance complexity and performance. Following this, the final step of the SE-NET, the scaling operation is executed:


(3) $${\tilde{x}}_{c}=F_{\text{scale}}\left(u_{c},s_{c}\right)=s_{c}\cdot u_{c}$$

In the formula, $\tilde{x}$ represents the feature map processed by the SE-module, where $u_{c}$ denotes the *c*th channel of the original feature map. $s_{c}$ represents the weight of the *c*th channel generated by the excitation operation. Ultimately, this scaled feature map $\tilde{X}$ is passed on to the next layer of the network. In this way, SE-NET dynamically adjusts the importance of different channels to enhance the network’s capability in representing features.

### 2.6 Weight GATs for prediction

After constructing the protein contact graph and obtaining the node features, the proposed method applies the GAT (Veličković *et al.* 2017). The attention mechanism introduced by GAT enables the model to focus on the most important node relationships, which improves the accuracy and interpretability of the prediction. Specifically, in the GAT layer, the input is a set of node features, which represent the residues of the protein, and the number of nodes is N. For each residue *i*, the similarity coefficient between it and each of its neighbor residues ($j\in N_{i}$) is calculated one by one:


(4) $$e_{ij}=a([Wh_{i}∥Wh_{j}]),j\in N_{i}$$

In the model, *a* represents a learnable attention mechanism function that maps the features of nodes *i* and *j* to a real number, signifying the importance of the edge $e_{ij}$. $h_{i}$ and $h_{j}$ denote the feature vectors of the nodes, and W stands for the weight matrix. Subsequently, we employ a shared attention mechanism to perform self-attention on the nodes, calculating the attention coefficients for the node and its neighbors. The attention coefficients are then normalized as follows:


(5) $$\alpha_{ij}=\frac{\;\text{exp}(\text{LeakyReLU}(e_{ij}))}{\sum_{k\in N_{i}}\;\text{exp}(\text{LeakyReLU}(e_{ik}))}$$

After obtaining the normalized attention coefficients, it is essential to calculate the linear combination of the corresponding features. Once passing through a nonlinear activation function, the final output feature vector for each node is as follows:


(6) $$h_{i}^{\prime }=\sigma \left(\frac{1}{K}\sum_{k=1}^{K}\sum_{j\in N_{i}}\alpha_{ij}^{k}W^{k}h_{j}\right)$$

Here, the number of heads in the multihead attention mechanism is 4.

### 2.7 Tackling data imbalance issues with label transfer strategy

**Figure 2.**
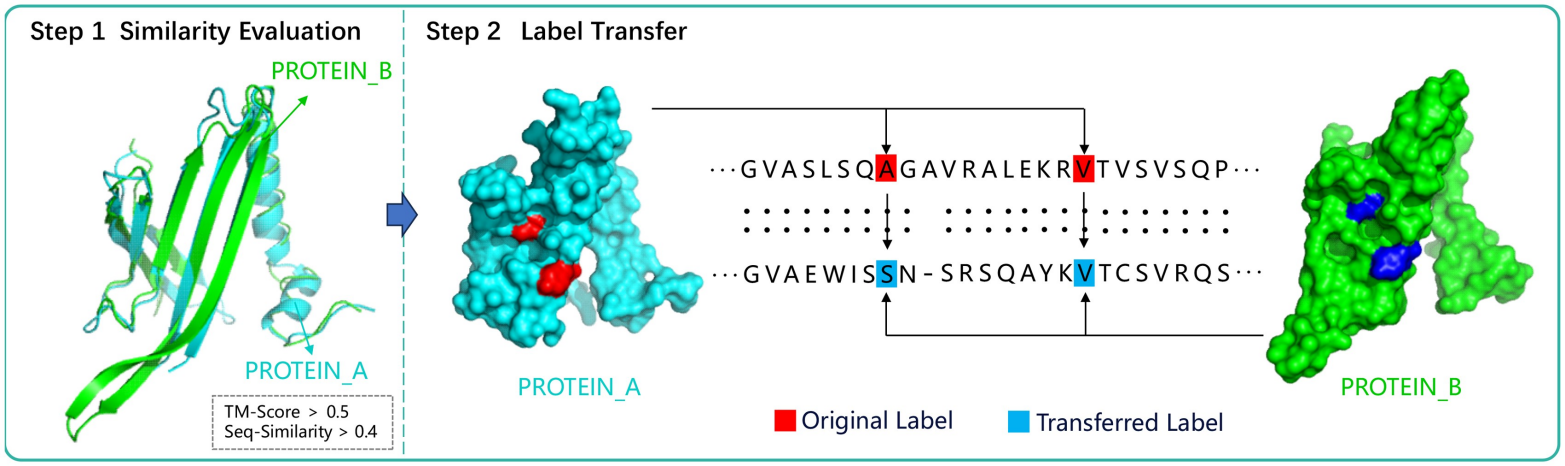
The process of label transfer. Firstly, the sequence similarity and structural similarity of the protein chains are calculated. Chains with a sequence similarity greater than 0.4 and a TM-score greater than 0.5 meet the criteria for label transfer. Subsequently, we transfer the annotations of the hotspot residues from chains in the same cluster to the chain with the maximum number of residues, to fully preserve the information of the hotspot residues.

In study of hotspot residue prediction in protein–RNA complexes, we encountered a significant challenge due to data imbalance: hotspot residues are considerably less frequent than nonhotspot residues. This imbalance can adversely affect the prediction accuracy of our model. To mitigate this issue, we employ a label transfer method to augment the original dataset. This approach is predicated on the assumption that proteins from different organisms can exhibit functional similarities when they share high sequence and structural similarity, despite potential differences in biological functions (Xia *et al.* 2021). Initially, the proposed method assess protein chains by calculating sequence similarity with BLAST (Altschul *et al.* 1997) and structural similarity using TM-align (Zhang and Skolnick 2005), selecting chains that exhibit a sequence similarity greater than 0.4 and a TM-score above 0.5. Subsequently, it is essential to transfer hotspot residue annotations from chains within the same cluster to the chain that possesses the most residues, ensuring maximal retention of hotspot information. The label transfer process is depicted in Fig. 2. After the label transfer, the data needs to be rigorously processed, which includes verifying the experimental validation of the transferred hotspot residues. The findings (Katsamba *et al.* 2002, Belashov *et al.* 2018) indicate that a majority of the transferred residues have been confirmed as hotspots in the literature, validating the effectiveness of our augmentation method. As a result, the data on hotspot residues increased by 9% after deduplication.

## 3 Results and discussion

### 3.1 Five-fold cross-validation performance assessment

**Figure 3.**
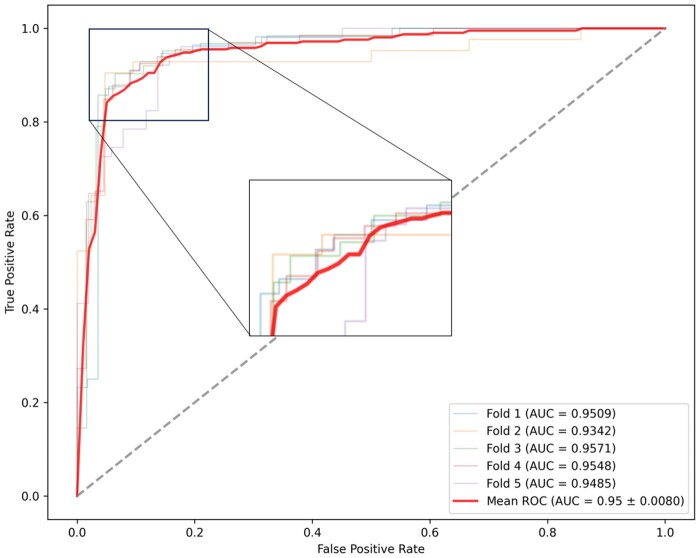
Performance of DeepHotResi on five-fold cross-validation.

The performance of the proposed model is evaluated using five-fold cross-validation (five-fold CV), a robust statistical method that assesses the generalization ability of a model. In five-fold CV, the dataset is divided into five subsets. The model is trained on four of these subsets and tested on the remaining one. This process is repeated five times, ensuring each subset serves both as a training and a testing set. The model’s performance was assessed through five metrics, including: area under the curve (AUC), sensitivity (SEN), specificity (SPE), precision (PRE), F1-score, and Matthews correlation coefficient (MCC). In five-fold CV, DeepHotResi achieves the highest performance: AUC of 0.956, SEN of 0.957, SPE of 0.903, F1-score of 0.912, and MCC of 0.755. These results highlight the model’s strong capability in accurately identifying hotspot residues in protein–RNA complexes. Figure 3 illustrates the area under the ROC curve for these methods on the dataset, providing a visual representation of our model’s superior performance.

### 3.2 Comparison with baseline methods

To evaluate the performance of DeepHotResi, we compared seven baseline models with our method: support vector machine (SVM) (Hearst *et al.* 1998), random forest (RF) (Breiman 2001), XGBoost (Chen and Guestrin 2016), deep neural network (DNN) (LeCun *et al.* 2015), graph convolutional network (GCN) (Kipf and Welling 2017), Graphformes (Veličković *et al.* 2017) and GraphGPS (Rampášek *et al.* 2022), respectively. The input features for SVM, RF, XGBoost, and DNN include PSSM, HMM, DSSP, and embeddings generated by ESM-2. In contrast, GCN, Graphformes, and GraphGPS, as graph neural network methods, additionally incorporate the protein contact map along with these feature representations. For SVM, RF, and XGBoost, we adopt the default hyperparameters available in the sklearn library, while the hyperparameters for DNN, GCN, Graphformes, and GraphGPS are selected based on experimental results. Specifically, DNN employs a hidden layer dimension of 32, GCN is configured with one GCN layer of dimension 32. Graphformes and GraphGPS are set with eight attention heads. The optimizer for DNN, GCN, Graphformes, and GraphGPS is Adam. The results are presented in Table 2. DeepHotResi achieves the highest scores in SPE, PRE, F1-score, and AUC. These results underline the effectiveness of DeepHotResi in addressing the challenges posed by data imbalance and the complexity of hotspot residue prediction in protein–RNA complexes.

**Table 2.** Results of feature ablation experiment on five-fold cross-validation.

Methods	SEN	SPE	PRE	F1	MCC	AUC
SVM	0.735	0.805	0.789	0.760	0.540	0.770
RF	0.755	0.797	0.787	0.767	0.553	0.776
XGB	0.752	0.741	0.744	0.746	0.492	0.746
DNN	0.848	0.807	0.821	0.830	0.658	0.828
GCN	0.942	0.642	0.839	0.888	0.768	0.921
Graphformes	0.953	0.812	0.835	0.890	**0.773**	0.941
GraphGPS	**0.986**	0.661	0.744	0.848	0.683	0.939
DeepHotResi	0.957	**0.903**	**0.872**	**0.912**	0.755	**0.956**

Note: The best performance for each metric is marked in bold.

### 3.3 Performance comparison on the independent dataset

To validate the performance of DeepHotResi, we conduct a comparative analysis against several established methods, including XGBPRH, PrabHot, HotSPRing, and SREPRHot. During comparison, each method is evaluated on the same test set to ensure fairness in the comparison. For the purposes of comparison, all methods are evaluated under the same threshold condition of 1.0 kcal/mol for defining hotspots, with the exception of SREPRHot, which uses a threshold of 2.0 kcal/mol. The results in Table 3 demonstrate that DeepHotResi consistently outperforms the other methods across all metrics: achieving an SEN of 0.926, SPE of 0.862, precision of 0.862, F1-score of 0.893, and an MCC of 0.755. Notably, our model excels particularly in specificity and precision, significantly surpassing the comparative methods. This indicates DeepHotResi’s exceptional ability to accurately identify nonhotspot residues. Additionally, DeepHotResi exhibits superior performance in the AUC metric, scoring 0.934, which was higher than PrabHot (0.817), HotSPRing (0.658), XGBPRH (0.868), and SREPRHot (0.829). These results underscore the proposed model’s excellent capability in predicting hotspot residues in protein–RNA complexes, confirming its effectiveness and reliability in this critical area of biomolecular research.

**Table 3.** Performance comparison of DeepHotResi with other approaches on the independent dataset.

Method	SEN	SPE	PRE	F1	MCC	AUC
XGBPRH	0.909	0.733	0.833	0.870	0.661	0.868
PrabHot	0.793	0.655	0.697	0.742	0.453	0.817
HotSPRing	0.655	0.552	0.604	0.633	0.258	0.658
SREPRHot	0.900	0.792	0.474	0.621	0.557	0.829
DeepHotResi	**0.926**	**0.862**	**0.862**	**0.893**	**0.755**	**0.934**

Note: The best performance for each metric is marked in bold.

### 3.4 Pretrained language model features improve model performance

The impact of ESM-2 pretrained language model’s sequence embeddings is investigated through a feature ablation experiment. The experiment designates “w/o ESM-2” involves excluding the ESM-2-generated sequence embeddings, relying solely on DSSP, HMM, and PSSM features. Likewise, “w/o HMM”, “w/o DSSP”, and “w/o PSSM” indicate the exclusion of HMM, DSSP, and PSSM features, respectively. The evaluation metrics included SEN, SPE, PRE, F1-score, MCC, and AUC.

The results displayed in Table 4 reveal the impact of different feature sets on the model’s performance. With all features integrated, the model achieves the best comprehensive performance in SPE, PRE, F1-score, and AUC. However, excluding the ESM-2 sequence embeddings significantly deteriorates performance. The removal of DSSP, PSSM, or HMM features also leads to a decline in performance, albeit to a lesser extent than removing ESM-2 embeddings. This decline is particularly noticeable in SPE and MCC metrics, underscoring the critical contribution of ESM-2 to the model’s efficacy. Although the sensitivity has slightly decreased after adding ESM-2 embeddings, other metrics have significantly improved. Therefore, we believe that it is meaningful to sacrifice a small amount of sensitivity in exchange for greater improvements in other performance aspects. In summary, the sequence representations provided by the ESM-2 pretrained language model are crucial for enhancing the model’s performance, more so than any single traditional feature set.

**Table 4.** Results of ablation experiment on five-fold cross-validation.

Feature	SEN	SPE	PRE	F1	MCC	AUC
w/o ESM-2	0.978	0.028	0.503	0.667	0.049	0.698
w/o DSSP	0.945	0.787	0.816	0.876	0.742	0.934
w/o HMM	**0.985**	0.755	0.800	0.883	0.760	0.936
w/o PSSM	0.942	0.642	0.839	0.888	**0.768**	0.921
All features	0.957	**0.903**	**0.872**	**0.912**	0.755	**0.956**

Note: The best performance for each metric is marked in bold.

### 3.5 Impact of ESM-2 pre-trained language model

**Figure 4.**
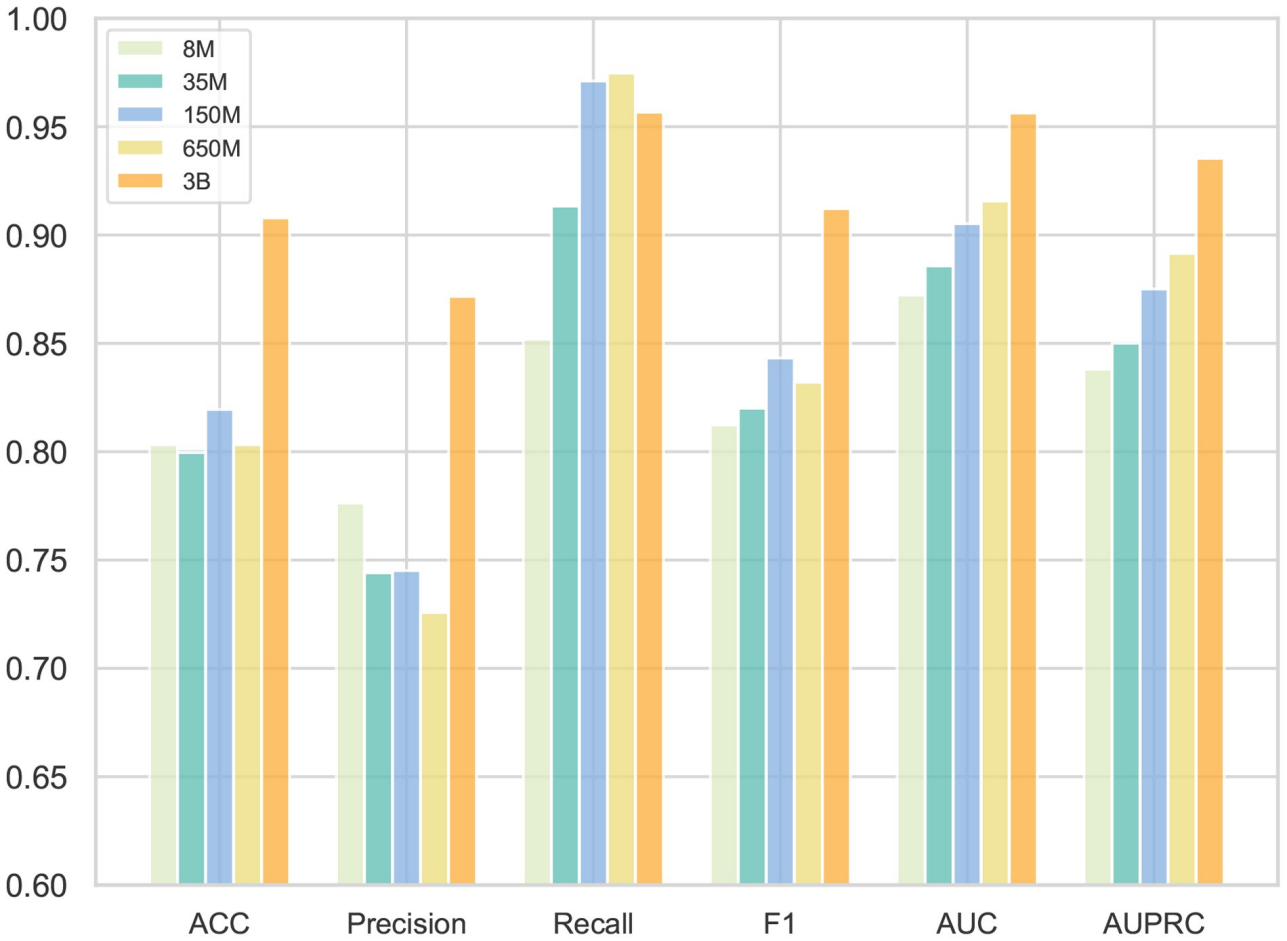
Results of different parameter scales on the feature generated by protein language models.

In this analysis, we examine the influence of various parameter sizes in ESM-2 pretrained protein models on the extracted amino acid sequence features. Five ESM-2 pre-trained models with parameter sizes of 3B, 650M, 150M, 35M, and 8M are utilized to derive these features. The experimental results reveal a clear trend: larger parameter sizes generally result in more accurate predictions. This indicates that ESM-2 models with more substantial parameters can more effectively capture the complex nuances of amino acid sequences, enhancing their ability to comprehend and predict protein structure and function. Specifically, the model with 3 billion parameters significantly outperforms the 8 million parameters model, illustrating a direct correlation between increased model size and enhanced feature extraction capabilities. This improvement is manifested in higher AUC values among other metrics. Figure 4 depicts the performance differences across ESM-2 models of varying sizes, highlighting their respective accuracy, precision (also known as SEN), F1-score, AUC, and area under the precision–recall curve metrics.

### 3.6 Attention contribution and visualization

**Figure 5.**
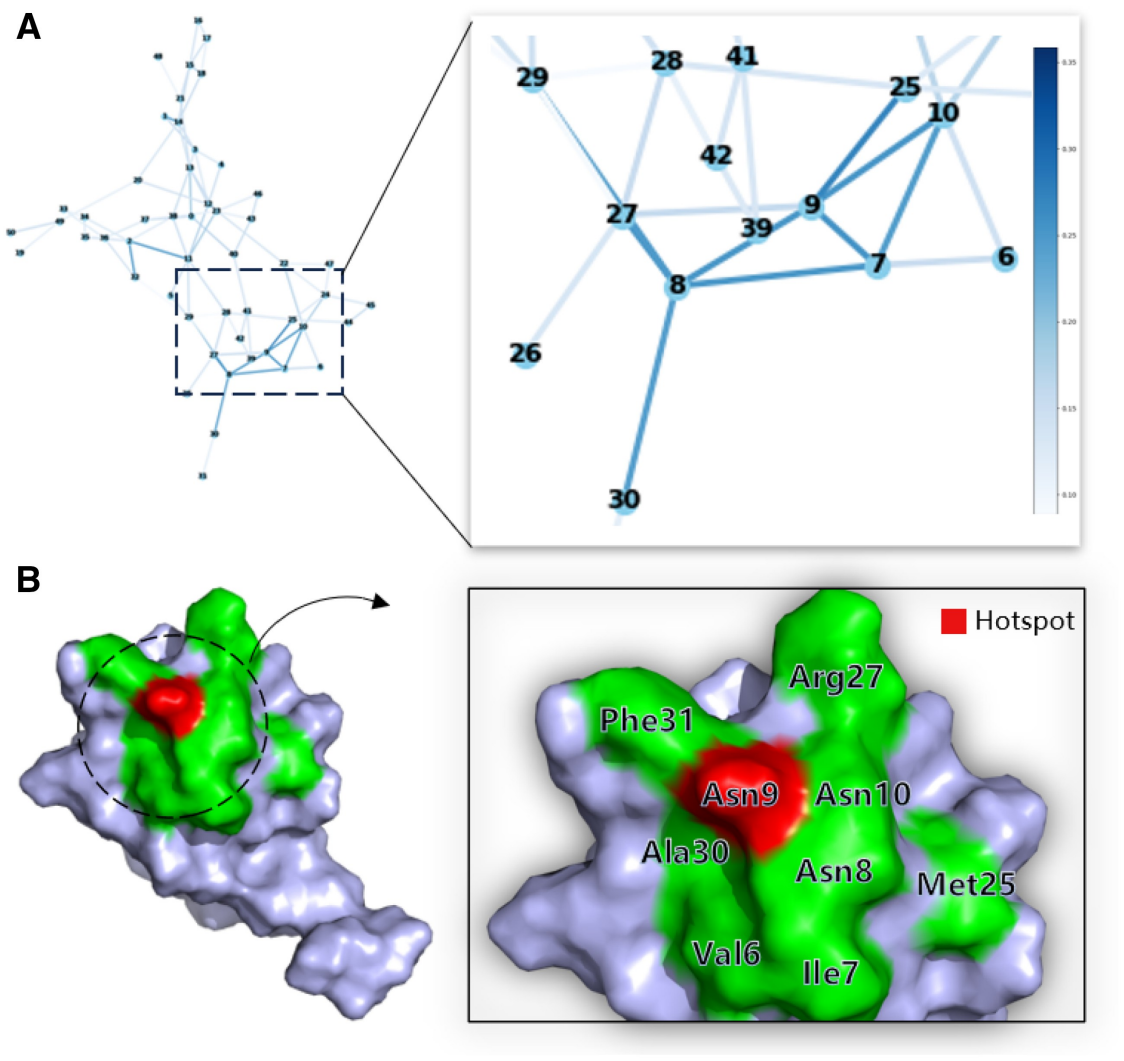
(A) Attention weight map for the protein 1L1C_A. This map utilizes a gradient of blue shades, ranging from light to dark, to quantify the attention weights at the contact points, with darker shades representing higher weight values. (B) 3D structural visualization of the protein residues with higher attention weights, highlighting hotspot residues in red to denote their significant contribution to the stability of the complex, while other residues are depicted in blue to indicate their relatively lower influence.

To demonstrate the effectiveness and interpretability of the proposed model in predicting hotspot residues within protein–RNA complexes, the attention contributions of the model is visualized. This approach can intuitively highlight the residues that the model focuses on during prediction, providing insight into their role in the decision-making process. We select the complex of the LicT bacterial antiterminator protein with its RNA target (PDB ID: 1L1C) (Yang *et al.* 2002) as a case study to visualize the attention weights allocated by our model. In the contact map visualization, residues such as Val6, Ile7, Asn8, Asn9, Asn10, Met25, Arg27, Ala30, and Phe31 exhibit higher attention weights, with Asn9 standing out as particularly prominent. Notably, Asn9 has been experimentally validated as a hotspot residue (Hübner *et al.* 2011), as shown in Fig. 5A.

The variation in attention scores suggests that residues surrounding the hotspot residues may play a key role in the protein’s functional activity. Previous studies have indicated that hotspots are often geometrically encased by contact residues, which are structurally less prominent and tend to be hydrophilic and partially solvated, forming an “O-ring” topology (Bogan and Thorn 1998, Thorn and Bogan 2001, Li and Liu 2009). To further investigate this, the spatial proximity of these residues are examined by PyMOL for 3D visualization of the hotspot residues and their surrounding environment, as depicted in Fig. 5B. The high-weight residues identified by the proposed model surround the experimentally validated hotspot, reinforcing the model’s ability to recognize hotspot residues spatially. This also highlights the model’s potential to capture the complexities of protein–RNA interactions.

These results demonstrate that our model accurately identifies key amino acid residues and their functional relevance, consistent with known biological data. The visualization of attention mechanisms provides a novel interpretive framework for understanding interactions, offering valuable insights for future biological research and drug design.

### 3.7 Impact of SE-module

The proposed model incorporates the SE-module to enhance the feature representation of residues. To evaluate its impact, we conduct ablation experiments by removing the SE-module from the model. We then train and test the model on the same dataset without the SE-module. As shown in Table 5, the model with the SE-module outperforms the one without it, highlighting that the SE-module helps the model focus more on key features while minimizing noise interference.

**Table 5.** Comparison of results with and without SE-module in five-fold cross-validation.

Feature	SEN	SPE	PRE	F1	MCC	AUC
w/o SE-module	0.853	0.042	0.513	0.673	0.119	0.697
w/SE-module	**0.957**	**0.903**	**0.872**	**0.912**	**0.755**	**0.956**

Note: The best performance for each metric is marked in bold.

### 3.8 Case study

**Figure 6.**
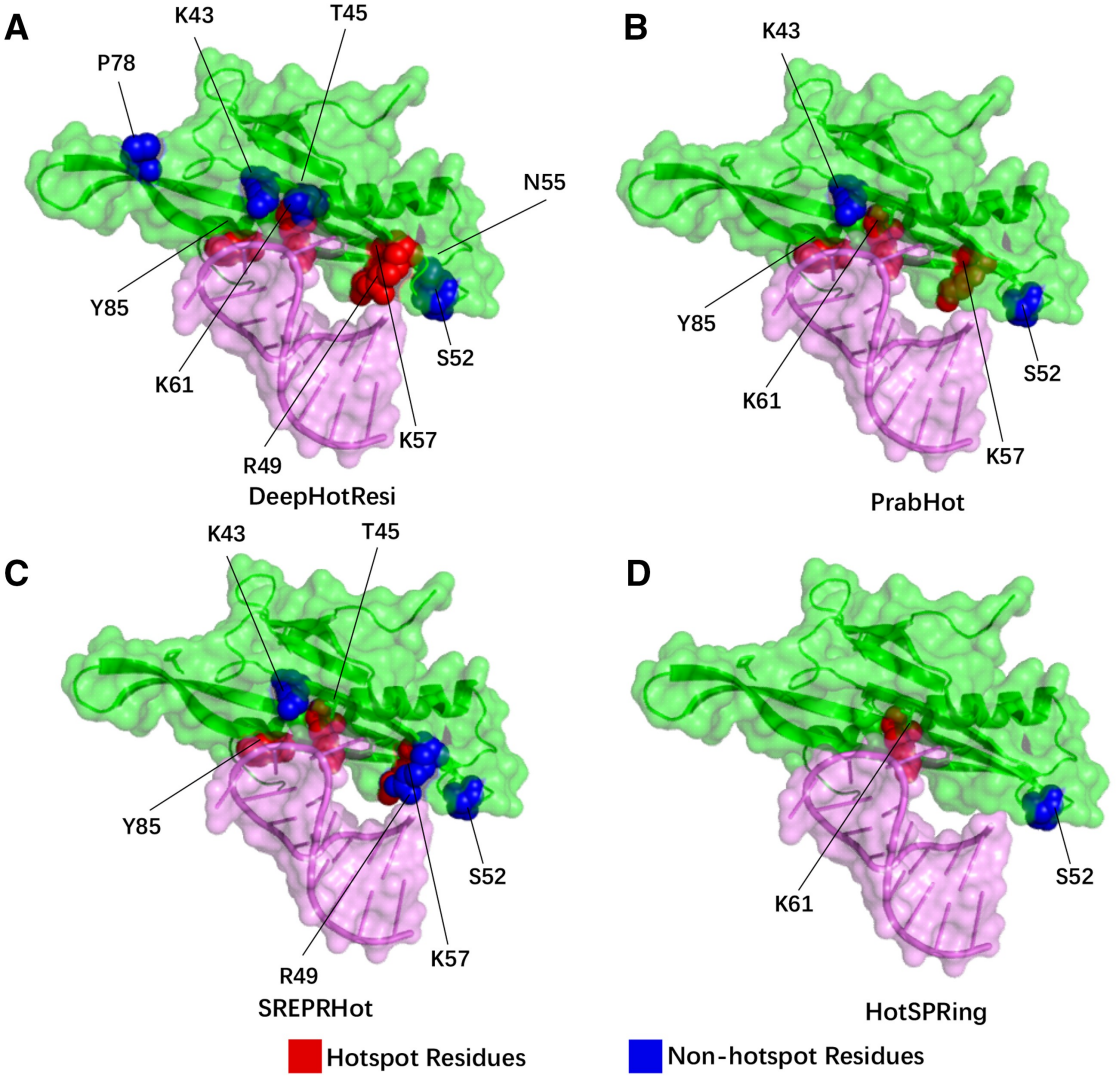
The hotspot residues identified by DeepHotResi (A) compared with the methods of PrabHot (B), SREPRHot (C), and HotSPRing (D) for predicting hotspot residues. The green surface denotes the protein chain of MS2 (PDB ID: 1ZDI, chain A), while the purple surface represents the RNA chain of MS2 (PDB ID: 1ZDI, chain R). Red color indicates residues experimentally validated as hotspots and correctly predicted as hotspots. Blue color indicates residues experimentally validated as nonhotspots and correctly predicted as nonhotspots.

To further investigate the predictive capabilities of DeepHotResi, we conduct a case study of the recombinant MS2 capsid protein–RNA complex (PDB ID: 1ZDI, Chain A). Recombinant MS2 capsid proteins form icosahedral viral capsids that protect viral nucleic acids and specifically bind to a unique site on RNA, which contains a stem-loop structure with the start codon of the viral replicase gene. The binding serves as a translational repressor (Valegård *et al.* 1997). In the case of this complex, alanine scanning experiments identified six nonhotspot residues (K43, T45, S52, N55, T59, P78) and four hotspot residues (R49, K56, K61, Y85). Through DeepHotResi method, we successfully predict all the hotspot residues and five nonhotspot residues (K43, T45, S52, N55, P78), as shown in Fig. 6A. PrabHot (Fig. 6B), HotSPRing (Fig. 6C), and SREPRHot (Fig. 6D) only correctly predict 3, 2, and 1 hotspot residues, respectively.

## 4 Conclusion

Protein–RNA interactions play a pivotal role in the molecular mechanisms and regulatory networks within cells. Within these complexes, certain residues, known as “hotspot residues,” are crucial for maintaining the stability and functionality of protein–RNA interactions. Investigating these hotspot residues not only enhances our understanding of disease mechanisms but also aids in the development of targeted therapies.

To address this challenge, this study develops a deep learning-based model and constructs a novel dataset, five times larger than existing datasets, to train the model. To mitigate the challenge of sparse hotspot data, we employ label transfer for data augmentation. The proposed model requires only the protein sequence as input and leverages the pre-trained ESM-2 model to predict three-dimensional structural information, subsequently constructing a contact map. Then, the proposed method extracts features, including secondary structure information from DSSP, sequence features from HMM, evolutionary data from PSSM, and residue embeddings from ESM-2. These features are integrated with SE-NET used for feature recalibration to enhance representation, and GAT is employed for hotspot prediction.

The experimental results demonstrate that the proposed method can efficiently and reliably identify hotspot residues in protein–RNA complexes. While the proposed model significantly outperforms existing state-of-the-art methods, there is still room for improvement. Current approaches focus primarily on protein features, often neglecting the critical contributions of RNA. In future work, we will explore incorporating RNA sequence and structural features, as well as protein and RNA binding site information. Additionally, given the limited data on hotspot residues in protein–RNA complexes, employing transfer learning and pretrained models could further enhance prediction accuracy and generalizability.

## Author Contributions

Siyuan Shen (Conceptualization, Data curation, Visualization, Methodology, Writing—original draft, Writing—review & editing, Formal analysis, Validation), Jie Chen (Software, Methodology), Zhijian Huang (Software, Writing—review & editing), Yuanpeng Zhang (Writing—original draft), Ziyu Fan (Writing—original draft), Yuting Kong (Writing—original draft), and Lei Deng (Conceptualization, Supervision, Resources, Funding acquisition, Writing—review & editing)

## Supplementary Material

btaf197_Supplementary_Data

## Data Availability

The source code and dataset are available at https://github.com/Q1DT/DeepHotResi.
