# Supplementary material for: Precise prediction of hotspot residues in protein–RNA complexes using graph attention networks and pretrained protein language models
Source: Bioinformatics. 2025 Jul 15;41(Suppl 1):i466–74. doi: 10.1093/bioinformatics/btaf197 (PMC12261489; doi:10.1093/bioinformatics/btaf197)
Supplement: btaf197_Supplementary_Data [file btaf197_supplementary_data.pdf]

Precise Prediction of Hotspot Residues in Protein-RNA Complexes Using Graph Attention Networks and Pre-trained Protein Language Models

Siyuan Shen[1], Jie Chen[12], Zhijian Huang[1], Yuanpeng Zhang[2], Ziyu Fan[1], Yuting Kong[3], and Lei Deng[1,2, *]

[1] School of Computer Science and Engineering, Central South University, Chang Sha, China. E-mail: leideng@csu.edu.cn
[2] School of software, Xinjiang University ,Urumqi China.
[3] School of Information Engineering, Xinjiang Institute of Engineering , Urumqi, China.

Supplementary Material

A. Dataset

The dataset organized in this study is shown below.

Table S1: The dataset of protein-RNA complexes

**Training dataset**	1A1T	1ASY	1C9S	1DFU	1JBS	1K8W	1L1C	1N78
	1O0B	1QFQ	1RPU	1T0K	1TTT	1WHQ	1YTU	1YVP
	1ZH5	2A1R	2A8V	2BX2	2ERR	2ESE	2IX1	2KG0
	2M8D	2MQO	2MQQ	2PJP	2XGJ	2XS2	2Y8W	2ZI0
	2ZKO	2ZZM	3EQT	3GPQ	3K49	3L25	3MOJ	3OL6
	3QSU	3RC8	3RW6	3SN2	3U4M	3V74	3VYX	3WBM
	4AQ7	4BS2	4CSF	4ED5	4ERD	4F3T	4HOT	4I67
	4L8H	4MDX	4NGB	4NKU	4O26	4OI0	4OOG	4PMW
	4Q9Q	4QI2	4QU7	4R3I	4R8I	4RCJ	4YVI	5AWH
	5DET	5DNO	5E3H	5ELH	5ELK	5GXH	5IP2	5KAL
	5M3H	5UDZ	5WWT	5WWX	5Y58	5Z98	6ALG	6CMN
	6D12	6G2K	6HYU	6LVR	6PZQ	6SO9	6WLH	7ACT
	7LD5	7YR6	7ZEW					
**Independent testing dataset**	1FEU	1WNE	1ZDI	2KXN	2XB2	3AM1	3UZS	3VYY
	4CIO	4G0A	4JVH	4NL3	5EN1	5EV1	5HO4	

[1] *Corresponding author

B. Input of Related Models and DeepHotResi

We classify the features into five categories: sequence features (e.g., position-specific scoring matrices, local structural entropy, conservation scores), structure features (e.g., secondary structure, energy scores), solvent exposure features (e.g., half-sphere exposure, residue depth, coordination number), residue interaction network features (e.g., betweenness centrality, closeness centrality, degree), and pre-trained embedding features derived from language models. These features collectively enhance the prediction of RNA-binding hot spots. The details on the input of recent methods and our DeepHotResi can be found in Table S2.

Table S2: The details on the input of recent methods.

Method	Sequence features	Structure features	Solvent exposure features	Residue interaction network features	Pre-trained embedding features
XGBPRH	✓	✓	✓	✓	×
PrabHot	✓	✓	✓	✓	×
HotSPRing	✓	✓	×	×	×
SREPRHot	✓	✓	×	×	×
DeepHotResi	✓	✓	×	×	✓

C. Evaluation Metrics

To evaluate the performance of the proposed model, we use evaluation metrics including SEN, SPE, PRE, F1-Score, MCC, ACC, AUC, and AUPRC. To clarify the meaning of these indicators, the following definitions are first made: True positives (TP) and true negatives (TN) represent the number of correctly predicted hotspot residues and non-hotspot residues, while false positives (FP) and false negatives (FN) represent incorrectly predicted hotspot residues and non-hotspot residues. The meaning of indicators is shown below.

1. SEN (Sensitivity), also known as recall, measures the proportion of actual positives that are correctly identified by the model.

$$\text{SEN} = \frac{TP}{TP + FN} \tag{1}$$

2. SPE (Specificity) measures the proportion of negative cases (non-hotspot residues) correctly identified by the model to all true negative cases.

$$\text{SPE} = \frac{TN}{TN + FP} \tag{2}$$

3. PRE (Precision) measures the proportion of true positive cases among the samples predicted by the model as positive cases.

$$\text{PRE} = \frac{TP}{TP + FP} \tag{3}$$

4. F1-score refers to the harmonic mean of precision and sensitivity, which comprehensively measures the balance performance of the model.

$$\text{F}_1 = \frac{2 \times \text{SEN} \times \text{PRE}}{\text{SEN} + \text{PRE}} \tag{4}$$

5. MCC (Matthews Correlation Coefficient) is an indicator that combines all classification results (TP, TN, FP, FN) and is suitable for imbalanced data.

$$\text{MCC} = \frac{TP \times TN - FP \times FN}{\sqrt{(TP + FP)(TP + FN)(TN + FP)(TN + FN)}} \tag{5}$$

6. ACC (Accuracy) measures the proportion of correctly predicted samples out of the total samples.

$$\text{ACC} = \frac{TP + TN}{TP + TN + FN + FP} \tag{6}$$

7. AUC (Area under the ROC curve) is the area under the ROC curve (with 1-specificity on the horizontal axis and sensitivity on the vertical axis), measuring the overall discriminative ability of the model.
8. AUPRC (Area under the precision-recall curve) evaluates the trade-off between precision and recall across different thresholds in binary classification tasks.

D. Impact of Dataset Size

A long-standing challenge in predicting hotspot residues in protein-RNA complexes is the insufficiency of data. In recent years, with the continuous growth of data volumes in the field of bioinformatics, we have the opportunity to utilize larger datasets to train more complex models, with the expectation of achieving more accurate predictions. This chapter aims to explore the specific impact of dataset size on the performance of models predicting hotspot residues in protein-RNA complexes. To systematically assess the impact of dataset size on model performance, we divide the dataset into four different sizes: 40%, 60%, 80%, and the complete 100%. Each dataset size is used to independently train the same prediction model to evaluate the impact of different data volumes on model performance. The experimental results, corresponding to the four different dataset sizes, show AUC values of 0.866, 0.871, 0.909, and 0.95, as illustrated in Fig. S1. These results indicate that the performance of the models improves significantly as the dataset size increases.

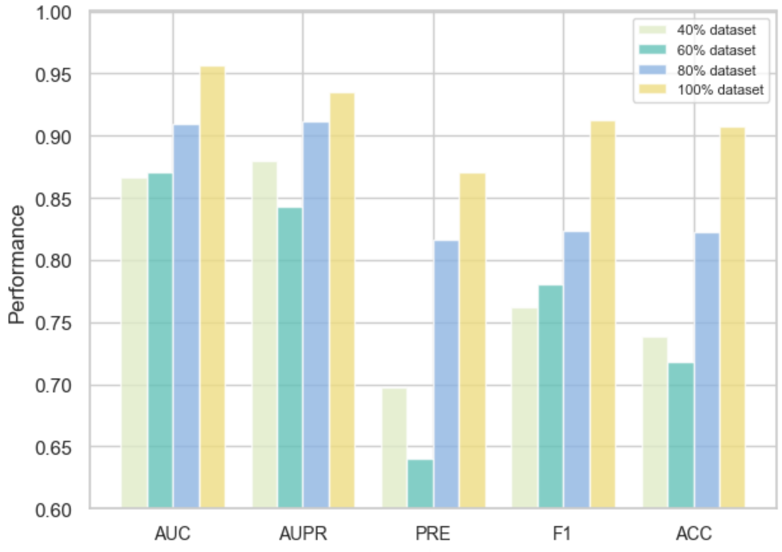

Fig. S1: Results of different dataset sizes.

E. Impact of Protein Contact Map

Contact maps of protein residues offer a translation and rotation-invariant topological representation of proteins. The selection of appropriate inter-residue distance cutoff values is crucial for constructing accurate contact maps, as it directly influences the definition of edges in the map and subsequent bioinformatics analyses. In this study, we reference a broad range of literature and

previous research [1,2,3], choosing 4 Å, 6 Å, 8 Å, 10 Å, and 12 Å as candidates for the residue distance cutoff values. The choice of these thresholds is based on their widespread application in the literature and the potential impact of different thresholds on the quality of contact maps. Experimental findings reveal that a cutoff value of 6 Å results in the model achieving an AUC value of 0.96, superior to the AUC scores obtained with cutoff values of 4 Å, 8 Å, 10 Å, and 12 Å, which are 0.9293, 0.9361, 0.9268, and 0.9163, respectively, as shown in Fig. S3. The model performs worst with a cutoff value of 12 Å.

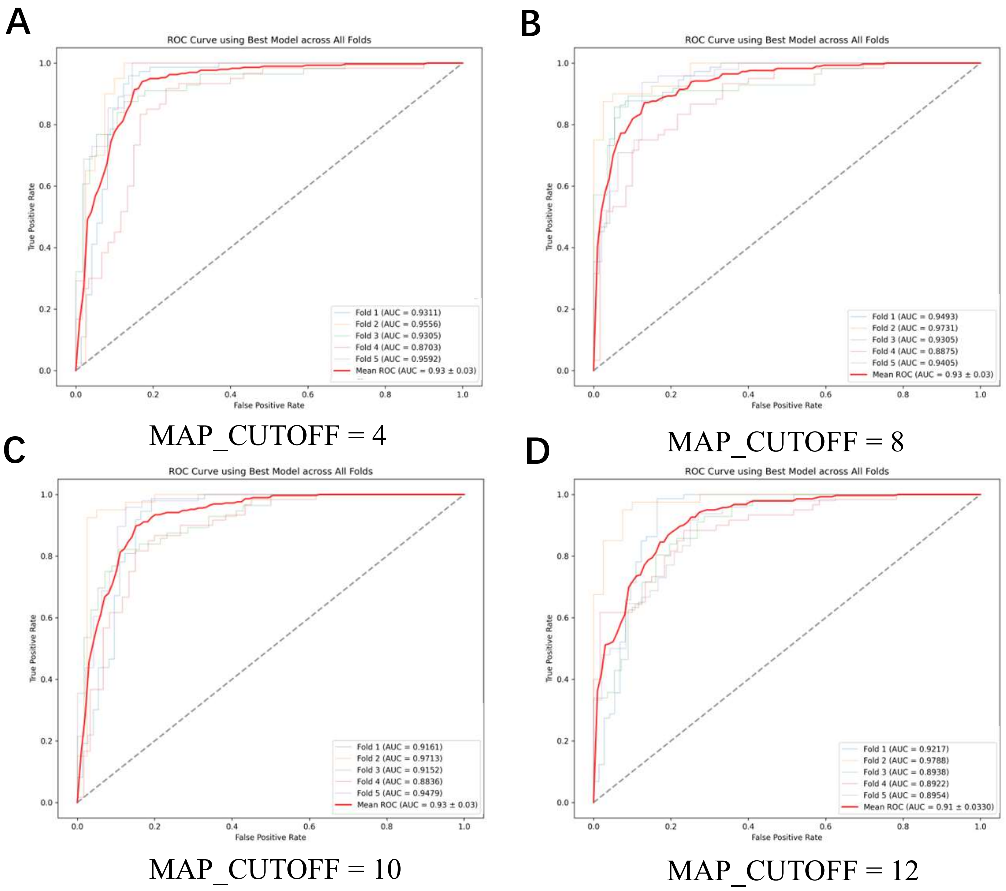

Fig. S2: Results of different protein contact map.

F. Impact of Transformer Head Numbers

GAT utilizes attention mechanisms to learn complex relationships between nodes. GAT employs multiple attention heads, each of which learns different attention weights, helping the model capture the diversity and complexity of the data more comprehensively. We evaluate the impact of different numbers of attention heads on the performance of the model. Fig. S4 shows the evaluation results of models with the number of attention heads ranging from 1 to 8. The results indicate that the model works best when the number of attention heads is 4. When the model has 1 to 4 attention heads, the prediction accuracy of the model improves as the number of attention heads increases.

However, the performance of the model gradually weakens when the number of attention heads exceeds 4.

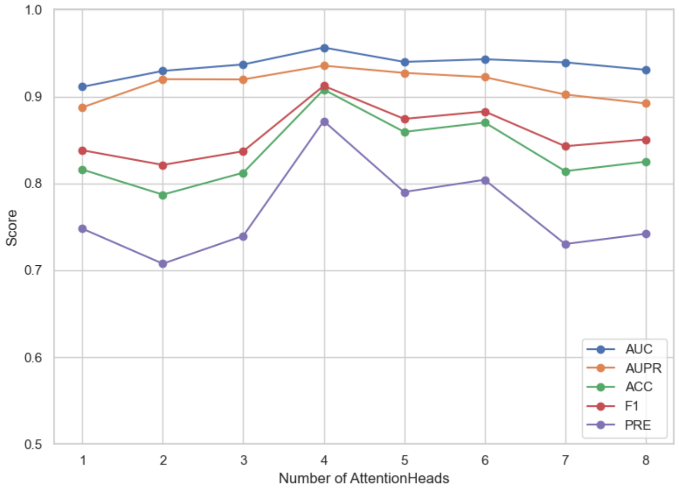

Fig. S3: Results of different transformer head numbers.

G. Impact of ESM-2

To validate the independent contribution of ESM-2 embeddings, we conducted three comparative experiments while keeping the model architecture entirely unchanged: 1) Baseline model (containing ESM-2 embeddings and other features), 2) Removed non-ESM-2 features while retaining only ESM-2 inputs, and 3) Eliminated ESM-2 embeddings while using only other original features. As shown in Table S3, the baseline model demonstrated significantly superior performance compared to both the ESM-2-only configuration and the configuration without ESM-2 embeddings.

Table S3: Results of ablation experiment on 5-fold cross-validation

Feature	SEN	SPE	PRE	F1	MCC	AUC
w/o ESM-2	0.978	0.028	0.503	0.667	0.049	0.698
Only ESM-2	0.928	0.780	0.808	0.864	0.715	0.938
w/ ESM-2	0.957	0.903	0.872	0.912	0.755	0.956

H. Impact of Label Transfer on Performance

With label transfer, we gained a total of 84 new labels. Some of the labels obtained through label transfer overlap with experimentally verified labels. As a result, after deduplication, we have 28 new hotspot residue label data. We compare the results of the model on an independent test set before and after removing the data added by label transfer. The experimental results are shown in Table S4. According to the table, it can be concluded that the model trained after label transfer and addition of the data set improved in SEN, SPE, PRE, F1 scores, MCC, AUC, and AUPRC.

Table S4: Performance comparison on the independent test set

Model	SEN	SPE	PRE	F1	MCC	AUC	AUPRC
Original	0.852	0.828	0.821	0.836	0.679	0.888	0.878
Transferred	0.926	0.862	0.862	0.893	0.755	0.934	0.929
Optimization effect	8.69%	4.11%	4.99%	6.82%	11.19%	5.18%	5.81%

I. Comparison between SE-NET and Transformer

We conducted comparative experiments to evaluate the performance of the model using Squeeze Excitation Network (SE-NET) and Transformer for feature recalibration. The experimental results are shown in Table S5. According to the table, under the same training configuration, the performance of the model using SE-NET is superior to the Transformer-based method. Therefore, we choose SE-NET for the feature recalibration module, effectively improving the model's ability to filter and focus on features. From this table, it can be seen that by using SE-NET method, SEN, SPE, PRE, F1, and AUC were improved by 3.13%, 9.72%, 3.81%, 3.40%, and 0.95%, respectively, achieving more accurate extraction of key features and enhancing the overall performance and generalization ability of the model.

Table S5: Comparison between SE-NET and Transformer

Method	SEN	SPE	PRE	F1	MCC	AUC
Transformer	0.928	0.823	0.840	0.882	0.755	0.947
SE-NET	0.957	0.903	0.872	0.912	0.755	0.956
Optimization effect	3.13%	9.72%	3.81%	3.40%	0.00%	0.95%

References

1. Guo, Z., Wu, T., Liu, J., Hou, J., Cheng, J.: Improving deep learning-based protein distance prediction in casp14. Bioinformatics **37**(19), 3190–3196 (2021)
2. Kukic, P., Mirabello, C., Tradigo, G., Walsh, I., Veltri, P., Pollastri, G.: Toward an accurate prediction of inter-residue distances in proteins using 2d recursive neural networks. BMC bioinformatics **15**, 1–15 (2014)
3. Wang, K., Zhou, R., Tang, J., Li, M.: Graphscoredta: optimized graph neural network for protein–ligand binding affinity prediction. Bioinformatics **39**(6), btad340 (2023)
